# Mathematical Modeling for an MTT Assay in Fluorine-Containing Graphene Quantum Dots

**DOI:** 10.3390/nano12030413

**Published:** 2022-01-27

**Authors:** Paulo C. Morais, Dieime C. Silva

**Affiliations:** 1 Genomic Sciences and Biotechnology Program, Catholic University of Brasília, Brasília 70790-160, Brazil; dieime07@gmail.com; 2 Institute of Physics, University of Brasília, Brasília 70910-900, Brazil; 3 Department of Physics, Federal University of Rondônia, Porto Velho 76801-059, Brazil

**Keywords:** Hill-inspired model, cell viability, MTT assay, graphene quantum dot, size-dependence

## Abstract

The paper reports on a new mathematical model, starting with the original Hill equation which is derived to describe cell viability (V) while testing nanomaterials (NMs). Key information on the sample’s morphology, such as mean size (〈s〉) and size dispersity (σ) is included in the new model via the lognormal distribution function. The new Hill-inspired equation is successfully used to fit MTT (3-(4,5-dimethylthiazol-2-yl)-2,5-diphenyltetrazolium bromide) data from assays performed with the HepG2 cell line challenged by fluorine-containing graphene quantum dots (F:GQDs) under light (400–700 nm wavelength) and dark conditions. The extracted “biological polydispersity” (light: $\langle s_{\mathrm{MTT}}\rangle =1.77\pm 0.02\;\mathrm{nm}$ and $\sigma_{\mathrm{MTT}}=0.21\pm 0.02$); dark: $\langle s_{\mathrm{MTT}}\rangle =1.87\pm 0.02\;\mathrm{nm}$ and $\sigma_{\mathrm{MTT}}=0.22\pm 0.01$) is compared with the “morphological polydispersity” ($\langle s_{\mathrm{TEM}}\rangle =1.98\pm 0.06\;\mathrm{nm}$ and $\sigma_{\mathrm{TEM}}=0.19\pm 0.03$), the latter obtained from TEM (transmission electron microscopy). The fitted data are then used to simulate a series of V responses. Two aspects are emphasized in the simulations: (i) fixing σ, one simulates V versus 〈s〉 and (ii) fixing 〈s〉, one simulates V versus σ. Trends observed in the simulations are supported by a phenomenological model picture describing the monotonic reduction in V as 〈s〉 increases ($V\sim p^{a}/{\left(s\right)}^{p-a}$; p and a are fitting parameters) and accounting for two opposite trends of V versus σ: under light (V~σ) and under dark (V~1/σ).

## 1. Introduction

The interest in conducting the mathematical modeling of biological data, particularly in vitro standard assays, has grown tremendously (by about two orders of magnitude) in the last five decades from a few peer-reviewed publications in the early seventies to a few hundred in recent years, as witnessed by the records of scientific data [1]. The benefits of this trend are multifaceted, ranging from a minimization in the use of cell lines up to helping the improved planning of all biological assays, with the aim to maximize resources and minimize replication [2]. A key crossing issue is the recent global reproducibility initiative, which has targeted the replication of selected published experiments in specific bio-related areas to further share the benefits with the scientific community and which aims to advance scientific progress while reducing the replication of work [3,4]. The present study is designed to make a contribution to the above-mentioned prospects, focusing on a comprehensive assessment of the mathematical models currently in use for handling and interpreting in vitro experimental data. More importantly, the paper aims to contribute to data analyses related to the involvement of nanomaterials (NMs) in the biological assay, particularly emphasizing the morphological characteristics represented by the mean size (〈s〉) and the size dispersity (σ) of the tested NM. Even before the “NANO” came along, in the mid-1990s, recognition of the size-dependent biological responses of polymeric nanoparticles was clearly stated [5]. In recent years, however, the number of publications devoted to the evaluation of the biological responses while using NMs interacting either with cells or biomolecules is increasing steeply [6,7,8,9,10,11,12,13,14,15,16].

Regarding the handling of in vitro data while exploring the ligand–receptor relationship, a milestone step in the field was put forward by Archibald Vivian Hill in 1910 [17]. In fact, this early publication of the 1922 Nobel Laureate (A.V. Hill) followed his first ever published paper on the antagonistic action of nicotine and curari molecules on a frog’s skeletal muscles [18]. The scheme used by Hill to derive what was coined the Hill equation was intended to explain the binding of oxygen (O_2_) onto a single hemoglobin (Hb) or clusters comprising *n*-molecules of hemoglobin [17]. In this particular case, a cluster comprising *n*-molecules of hemoglobin would provide up to *n*-sites for oxygen binding, with oxygen as the “ligand” and hemoglobin as the “receptor”. At the time, the experiments analyzed by Hill involved the saturation of Hb by O_2_ as the oxygen pressure increased. The remarkable quality of the experimental data fittings from different experimental sources using the derived Hill equation surprised A.V. Hill himself [17]. The same scheme and equation proposed by Hill [17], or schemes and equations inspired by this keystone model picture, have been used since the 1910 publication came along. In fact, the Hill scheme has been used to explain a huge diversity of in vitro data, among them the experiments that were planned to evaluate the response of a cell line (receptor) challenged by a bioactive compound (ligand). Particularly interesting is the Hill coefficient extracted from the application of the Hill equation while performing the fitting of experimentally related ligand–receptor data. Originally, the Hill coefficient (*n*) was taken as the number of ligands bound to a receptor. In recent years, publications reporting the use of the original Hill’s model or Hill-inspired models have continued to grow and increasingly involve the evaluation of the cytotoxicity of NMs.

Within the framework proposed by Hill [17], the main aspects and challenges of handling numerical data describing the receptor–ligand functional dependency have been emphasized by Vladimir Pliska [19]. Regarding receptor–ligand functional dependency, the cooperativity and allostery have been reviewed by Jan Krusek [20]. As pointed out by Krusek [20], it is worth mentioning the thermodynamic symmetry behind the receptor–agonist interaction modulated by the antagonist, which has already been observed in many systems: the entropy-driven receptor–agonist binding leads to enthalpy-driven receptor–antagonist binding and vice versa. A slight modification of the Hill equation was used by Mouton and Vinks [21] when analyzing the pharmacokinetic and pharmacodynamics of different antibacterials in in vitro and in vivo assays. In their analysis, the authors introduced the growth rate in addition to the kill rate, and thus were able to extract the stationary concentration (SC) plus the minimum inhibitory concentration (MIC) and the influence of the Hill coefficient over them. They found remarkable differences between SC and MIC for concentration-dependent antibacterials whereas for concentration-independent antibacterials, slight differences between SC and MIC were observed. The difference between SC and MIC is indeed a key point for assessing the post-antibiotic effect (PAE) in in vivo assays. Goutelle et al. [22] reviewed different aspects of the Hill equation and pointed out the huge variety of experimental data already analyzed by the Hill equation or approaches inspired in the Hill equation. For instance, muscle cells challenged with acetyl choline have been analyzed by Clark [23] and the pharmacokinetic–pharmacodynamic (PK–PD) describing the influence of the drug concentration dependence on the drug effect has been reported by Holford et al. [24], Mager et al. [25] and Csajka and Verotta [26]. The activity of antibiotics against microorganisms has been described by Zhi et al. [27] and Corvaisier et al. [28]; the aminoglycoside nephrotoxicity was analyzed by Giuliano et al. [29] and Rougier et al. [30]; the synergy and antagonism between two drugs in a pre-clinical study were discussed by Sperrin et al. [31]; the evaluation of the therapeutic index of a drug by balancing the dose–response plus the dose–toxicity was exemplified by Troche et al. [32]. Very recently, the Hill equation was used by Li et al. [33] to evaluate the cytotoxicity of fluorine-containing graphene quantum dots (F:GQDs) using the HepG2 cell line. The experiments were conducted in the dark and under visible light illumination in order to assess the potential photodynamic characteristics of the new fabricated NM. Enhanced photodynamic characteristics are key aspects related to the engineering of photosensitizer nanomaterials for application in photodynamic therapy (PDT), the latter requiring the production of a high density of reactive oxygen species (ROS) under illumination with a specific wavelength [34].

Graphene quantum dots (GQDs) represent a new class of carbon-based (carbon allotrope) zero-dimensional (0D) quantum dots (QDs) up to about three carbon monolayers in thickness and with a lateral dimension below 100 nm, thus implying a carriers’ quantum confinement in the two orthogonal directions (in-plane and out-of-plane) which can be controlled by their shape, number of carbon atoms, and edge termination (armchair and zigzag) [35]. These material systems present unique physical properties as a result of their electronic structure, which can be calculated by solving the corresponding Hamiltonian using the tight-binding formalism within the nearest neighbor’s approximation [36]. In addition to the unique electronic, magnetic and optical properties, GQDs have been widely explored in the biomedical area and are viewed as very promising candidates for different applications, such as in PDT [33,37] and biosensing for cancer diagnostics [38].

Despite the increasing interest in using NMs to perform in vitro assays which is aimed at supporting future applications in the biomedical area, and the already recognized influence of both NMs’ morphological parameters 〈s〉 and σ in the biological response, we are not aware of any proposal on how to include such key information either into the original Hill’s model or in Hill-inspired models. Therefore, this study aims to introduce a pioneering proposal on how to include the mean size and size dispersity of a NM into the analyses of in vitro assays. Using the model introduced in the present study, curve fittings of cell viability extracted from MTT assays will be presented. As a result of including the mean size and size dispersity into the herein proposed Hill-inspired model to describe the cell viability data, the concept of “biological polydispersity”, not yet reported, is introduced in the present study. Moreover, simulations starting from the curve fittings of cell viability extracted from the MTT assays will be explored to demonstrate the impact on the biological responses while including both the 〈s〉 and *σ* of a NM into the analysis of a standard bioassay. Additionally, a phenomenological model picture for the observed trends extracted from the simulations will be proposed. Indeed, the mean size of the tested F:GQDs is located in the extreme range of values smaller than 4 nm.

## 2. Materials and Methods

The first step in the presentation of the new Hill-inspired model, which includes the morphological characteristics (〈s〉 and σ) of NMs to account for in vitro tests, is related to the curve fitting of cell viability data assessed via MTT tests published in [33]. Therein, the MTT experiments were performed in duplicate and the statistical significance (*p*-value) was determined using the Student’s test (*p*-values < 0.05 mean the results are significant). The second step uses the introduced Hill-inspired model to perform simulations, starting with the fitted parameters extracted from the MTT tests handled in the first step. The study published in [33] reports MTT tests performed under two different conditions; the first with the used cell line (HepG2) kept in the dark and the second with the same cell line illuminated with an LED source (400–700 nm; 40 mW/cm^2^; 12 min). In [33], the collected cell viability data were curve-fitted using the original Hill equation [17], namely $V\left(D\right)=A-B\left(D^{n}/K+D^{n}\right)$, with *V*, *D*, *n*, *K*, *A* and *B* representing, respectively, the cell viability, the dose of fluorine-containing graphene quantum dots (tested sample), the cooperativity index, the binding constant between the tested sample and the cells, and the two scaling parameters (*A* and *B*). The two sets of cell viability data (under dark and under illumination) were refitted using the Hill-inspired model herein introduced, including the sample’s morphological characteristics, i.e., 〈s〉 and σ. In the introduced Hill-inspired model, the lognormal distribution function was used to describe the sample’s polydispersity profile. While handling the two sets of *V* versus *D* data (dark and illumination), using the Hill-inspired model, the mean size ($\langle s_{\mathrm{MTT}}\rangle$) and the size dispersity ($\sigma_{\mathrm{MTT}}$) were fitted and compared with the morphological parameters obtained from the TEM (transmission electron microscopy) micrographs, namely $\langle s_{\mathrm{TEM}}\rangle =1.98\pm 0.06$ nm and $\sigma_{\mathrm{TEM}}=0.19\pm 0.03$. Then, two full sets of fitting parameters (see Table 1) were generated while using the Hill-inspired model; one for the dark and another for the illumination condition. These two full sets of fitting parameters were used to simulate the results presented and discussed in this study. One set of simulated data explores the influence of increasing the mean size (〈s〉), in the range of 2.0 to 3.5 nm, while keeping the size dispersity fixed in $\sigma_{\mathrm{MTT}}=0.21$ or $\sigma_{\mathrm{MTT}}=0.22$ for illumination and dark conditions, respectively. The other set of simulated data explores the influence of increasing the size dispersity (σ), in the range of 0.25 to 0.40, while keeping the mean size fixed in $\langle s_{\mathrm{MTT}}\rangle =1.77\;\mathrm{nm}$ or $\langle s_{\mathrm{MTT}}\rangle =1.87\;\mathrm{nm}$ for illumination and dark conditions, respectively. Fittings and simulations were carried out using the Scientist™ MicroMath^®^ software commercialized by MicroMath Scientific Software (Salt Lake City, UT, USA).

## 3. The Mathematical Model

**Scheme 1:**
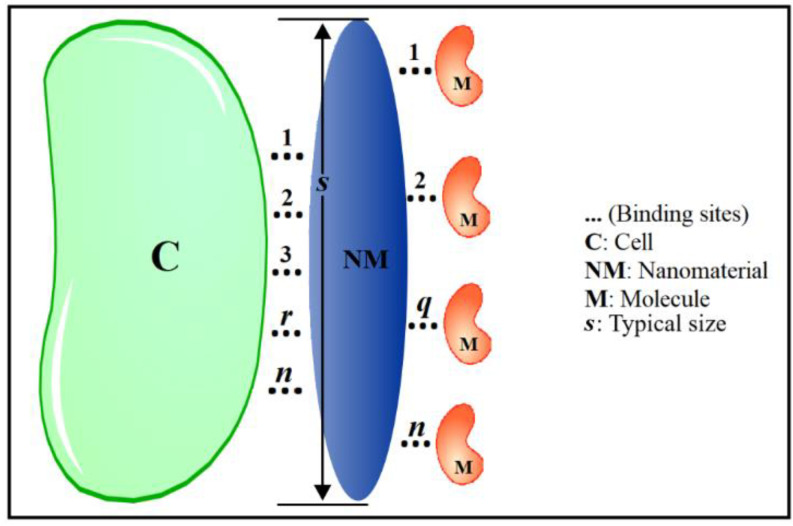
Schematic representation of the links (*r*) established between the nanomaterial (NM) and the cell (C) on the left-hand side of the panel, with *r* running from 1 up to *n*. Links between the nanomaterial (NM) and the biomolecules (M) are schematically shown on the right-hand side of the panel, with *q* running from 1 up to *n*. The typical size of the nanomaterial is described by *s*.

The derivation of the Hill equation [17] started with the equilibrium describing the binding of a certain number (*n*) of a particular ligand (L) to a receptor (R). Within the context of an NM, the Hill-inspired equation will be herein written as the binding of a certain number (*n*) of ligand sites located onto the surface of the NM (L@NM) to a particular number of receptors (R), the latter being for instance biomolecules (*q* binding molecules) or a cell membrane (*r* binding sites onto the cell). Scheme 1 shows the number of binding sites (*n*) at the NM’s surface and the number of receptors (*q*, *r*) in the two scenarios mentioned above (biomolecules-M or cell membrane-C). Then, for instance (q biomolecules), the equilibrium equation can be written as:

(1) $$qR+nL@\mathrm{NM}\;\begin{array}{c} k_{b}\\ ⇆\\ k_{f} \end{array}\;R_{q}{\left(L@\mathrm{NM}\right)}_{n},$$

with *k_f_* and *k_b_* meaning the forward and backward kinetic constants, respectively. In Equation (1), *n* is taken as the original Hill’s coefficient and interpreted as the number of binding sites at the NM’s surface, which are available for binding to *q* (or *r*) receptors.

Note that Equation (1) reduces to the original Hill equilibrium equation as *q* = 1, with L@NM equal to L. From the Guldberg and Waage’s law of mass action, the forward and backward kinetic constants *k_f_* and *k_b_* are related to the forward and backward reaction rates $v_{f}=k_{f}{\left[R\right]}^{q}{\left[L@\mathrm{NM}\right]}^{n}$ and $v_{b}=k_{b}\left[R_{q}{\left(L@\mathrm{NM}\right)}_{n}\right]$, respectively [39]. Therefore, in the thermodynamic equilibrium, the formation constant ($K_{F}$) is written as $K_{F}=\left[R_{q}{\left(L@\mathrm{NM}\right)}_{n}\right]/{\left[R\right]}^{q}{\left[L@\mathrm{NM}\right]}^{n}$. Note that the corresponding dissociation constant ($K_{D}$) is given by $K_{D}=1/K_{F}$. Similar to Hill’s original approach, the number of binding receptors (*B*: busy receptors) with respect to the number of total receptors, i.e., binding receptors (*B*) plus unbinding receptors (*E*: empty receptors) is described by the fraction $F=B/\left(B+E\right)=\left[R_{q}{\left(L@\mathrm{NM}\right)}_{n}\right]/\left(\left[R\right]+\left[R_{q}{\left(L@\mathrm{NM}\right)}_{n}\right]\right)$. Using the expression for $K_{F}$ above and the definition of the dissociation constant ($K_{D}=1/K_{F}$) into F=B/(B+E), one finds: $F={\left[R\right]}^{q}{\left[L@\mathrm{NM}\right]}^{n}/\left(K_{D}\left[R\right]+{\left[R\right]}^{q}{\left[L@\mathrm{NM}\right]}^{n}\right)$. Then, *F* can be simplified as:

(2) $$F\left(\left[L@\mathrm{NM}\right]\right)=\frac{{\left[L@\mathrm{NM}\right]}^{n}}{K+{\left[L@\mathrm{NM}\right]}^{n}},$$

with the unbinding constant (K) described by $K=K_{D}/{\left[R\right]}^{q-1}$. It is worth mentioning that as q→1, $K\to K_{D}$ and Equation (2) tends towards the original Hill equation. However, in the scenario involving an NM, *q* (or *r*) might be greater than unit (q, r>1). Nevertheless, it will be herein considered that the tested biomolecule (M) is uniform, meaning that the tested molecules bind equally (*q* is a constant for a given biomolecule binding to a given NM’s surface) onto the NM’s surface. Likewise, the tested cell (C) is also herein considered uniform, meaning that the tested cell offers identical numbers of binding sites (*r*) to the NM’s surface. Therefore, using the same biomolecule (fixed concentration) or the same cell line (fixed number of cells) in an experiment, meaning *q* (or *r*) is fixed, Equation (2) allows one to extract the *n* parameter from the *F* versus [L@NM] data. Alternatively, in another experiment, the *q* number in Equation (2), via $K=K_{D}/{\left[R\right]}^{q-1}$, can be assessed while fixing the [L@NM] and running *F* versus [R]. Importantly, the latter possibility is a novelty brought about in this Hill-inspired approach, providing extra information while testing a NM.

In order to introduce the morphological characteristics of the NM into the proposed Hill-inspired model, we should look at Scheme 1, from which it is obviously intuitive that *n* is expected to scale with the typical size (*s*) of the tested NM. Moreover, while testing a polydisperse NM one should account for the mean value of the parameter *n*, i.e., 〈n(s)〉, explicitly dependent on *s*. The first step in describing the size influence of the NM is to explicitly assume the size-dependence (*s*) of *n*, herein taken as the first-order approximation for n(s). Then, one should write $n\left(s\right)=n_{0}+\alpha \left(s-s_{0}\right)$, where $n_{0}$, α and $s_{0}$ are fitting parameters and $n\left(s\right)=n_{0}$ for $s=s_{0}$. Indeed, incorporating the size dependence (the NM’s morphology) into Equation (2) leads to Equation (3) below:

(3) $$F\left(s,\;\left[L@\mathrm{NM}\right]\right)=\frac{{\left[L@\mathrm{NM}\right]}^{n\left(s\right)}}{K+{\left[L@\mathrm{NM}\right]}^{n\left(s\right)}}.$$

The second step in the formulation of the Hill-inspired model is to find out a reasonable distribution function to describe the NM’s polydispersity. The literature is rich in describing polydisperse NMs using the lognormal distribution function (P(s)), i.e., $P\left(s\right)=\left[\exp\left(-2\sigma^{2}\right)/s\;\sigma \sqrt{2\pi }\right]\exp\left[-\ln^{2}\left(s/s\right)/2\sigma^{2}\right]$, where 〈s〉 and σ describe the mean size and the size dispersity, respectively [40,41,42,43]. The third and last step to include the size dependence of the NM into the proposed Hill-inspired model is to analyze the experimental *F* versus [L@NM] data using Equation (4) below:

(4) F([L@NM])=∫F(s,[L@NM])P(s)ds.

From the fitting of the experimental F versus [L@NM] data using Equation (4), one can extract a full set of six parameters, namely K, $n_{0}$, α, $s_{0}$, $\langle s_{\mathrm{MTT}}\rangle$, and $\sigma_{\mathrm{MTT}}\;$(see Table 1). While fitting (or simulating) the MTT data using Equation (4), the limits of the independent parameter s (for 〈s〉~3) in the integral runs from s=0.01 to s=10. Moreover, from the extracted parameters one can estimate the mean number (〈n〉) of binding sites at the NM’s surface, i.e., $\langle n\rangle =n_{0}+\alpha \left(\langle s\rangle -s_{0}\right)$. It is very important to state that it would be interesting to compare the “biological polydispersity” of the NM, herein represented by $\langle s_{\mathrm{MTT}}\rangle$ and $\sigma_{\mathrm{MTT}}$, with the morphological polydispersity (e.g., $\langle s_{\mathrm{TEM}}\rangle$ and $\sigma_{\mathrm{TEM}}$), the latter assessed using high resolution microscopy, such as transmission electron microscopy, scanning electron microscopy or atomic force microscopy [44,45,46]. The term “biological polydispersity” used here is a novelty and relates to how a cell line (C) or a biomolecule (M) probes (biological response) the NM’s mean size ($\langle s_{\mathrm{MTT}}\rangle$) and size dispersity ($\sigma_{\mathrm{MTT}}$). In the present context, the term “biological polydispersity” is for the first time introduced in the present study.

## 4. Results and Discussions

**Figure 1:**
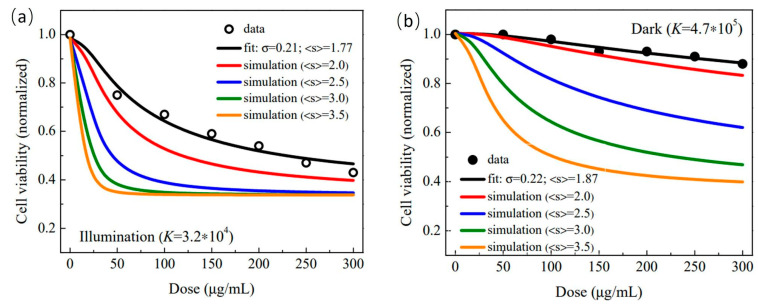
Cell viability (normalized) versus dose (μg/mL). (**a**) Open black symbols represent the cell viability obtained from the MTT data (HepG2 cells incubated with the F:GQD sample) collected under illumination while the solid black line is the best fit of the cell viability using Equation (4). Simulations of cell viability while fixing $\sigma_{\mathrm{MTT}}=$ 0.21 and increasing the mean size (〈s〉= 2.0, 2.5, 3.0 and 3.5 nm) are represented by solid colored lines. (**b**) Solid black symbols represent the cell viability obtained from the MTT data (HepG2 cells incubated with the F:GQD sample) collected in the dark while the solid black line is the best fit of the cell viability using Equation (4). Simulations of cell viability while fixing $\sigma_{\mathrm{MTT}}=$ 0.22 and increasing the mean size (〈s〉= 2.0, 2.5, 3.0 and 3.5 nm) are represented by solid colored lines.

**Figure 2:**
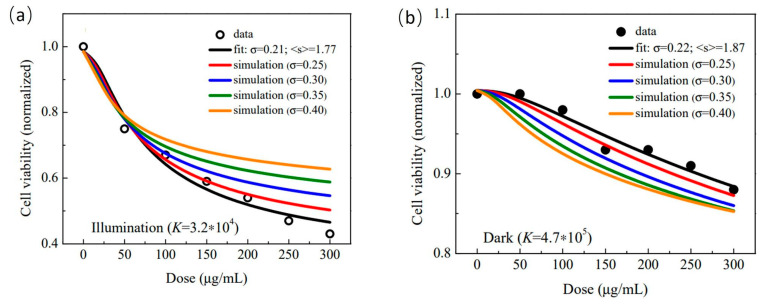
Cell viability (normalized) versus dose (μg/mL). (**a**) Open black symbols represent the cell viability obtained from the MTT data (HepG2 cells incubated with the F:GQD sample) collected under illumination while the solid black line is the best fit of the cell viability using Equation (4). Simulations of cell viability while fixing $\langle s_{\mathrm{TEM}}\rangle =1.77\;\mathrm{nm}\;$ and increasing the size dispersity (σ= 0.25, 0.30, 0.35 and 0.40) are represented by solid colored lines. (**b**) Solid black symbols represent the cell viability (HepG2 cells incubated with the F:GQD sample) collected in the dark while the solid black line is the best fit of the cell viability using Equation (4). Simulations of cell viability while fixing $\langle s_{\mathrm{TEM}}\rangle =1.87\;\mathrm{nm}$ and increasing the size dispersity (σ= 0.25, 0.30, 0.35 and 0.40) are represented by solid colored lines.

The impact of the NM’s morphological aspects on the biological response will be explored in this study using Equation (4), starting with the fitting parameters extracted from the analysis of the cell viability assay (MTT assay) performed with the HepG2 cell line incubated with the F:GQD, in the dark and under visible light illumination (see [33]). Importantly, the MTT test under visible light was performed to probe the capability of the F:GQD sample in generating ROS and therefore to explore its future application in PDT [33]. Symbols in Figure 1a,b represent the experimental values of the cell viability (V=1−F) under illumination (black open circles) and dark (black solid circles) conditions, respectively. Additionally, in Figure 1a,b, the black solid lines represent the best fit of the experimental data using Equation (4). Colored solid lines (red, blue, green, and orange) in Figure 1a,b represent simulations performed with the experimentally fitted parameters (K, $n_{0}$, α, $s_{0}$, and $\sigma_{\mathrm{MTT}}$) but with increases in the mean size (〈s〉) from 2.0 nm up to 3.5 nm, as indicated in the figures’ legends. Note the two full sets of fitted parameters (K, $n_{0}$, α, $s_{0}$, and $\sigma_{\mathrm{MTT}}$) collected in Table 1 and extracted from fitting the experimental cell viability data under illumination (Figure 1a) and dark (Figure 1b) conditions. Likewise, symbols in Figure 2a,b represent the experimental values of the cell viability under illumination and dark conditions, respectively. In Figure 2a,b, the black solid lines represent the best fit of the experimental data using Equation (4). Similarly, colored solid lines in Figure 2a,b represent simulations performed with the corresponding two full sets of experimentally fitted parameters (K, $n_{0}$, α, $s_{0}$, and $\langle s_{\mathrm{TEM}}\rangle$) but with increases in the size dispersity (σ) from 0.25 up to 0.40, as indicated in the figures’ legends. The two full sets of fitted parameters (K, $n_{0}$, α, $s_{0}$, $\langle s_{\mathrm{TEM}}\rangle$, and $\sigma_{\mathrm{MTT}}$) collected from the cell viability experiments (black open and solid circles in Figure 1 and Figure 2) and representative statistical values of the fittings (goodness-of-fit) for both experiments (dark and illumination) are collected in Table 1.

The first point to analyze in the two sets of parameters collected in Table 1 is the morphological characteristics of the F:GQD sample directly extracted from the TEM micrographs and indirectly obtained from the cell viability analysis using Equation (4). While the mean size assessed (revisited fitting) from the TEM data reported in [33] is $\langle s_{\mathrm{TEM}}\rangle =1.98\pm 0.06\;\mathrm{nm}$ ($\sigma_{\mathrm{TEM}}=0.19\pm 0.03$), the parameters extracted from the fittings of the MTT data (see Table 1) provide $\langle s_{\mathrm{MTT}}\rangle =1.77\pm 0.02\;\mathrm{nm}$ ($\sigma_{\mathrm{MTT}}=0.21\pm 0.02$) and $\langle s_{\mathrm{MTT}}\rangle =1.87\pm 0.02\;\mathrm{nm}$ ($\sigma_{\mathrm{MTT}}=0.22\pm 0.01$) for the MTT experiments conducted under illumination and dark conditions, respectively. Firstly, the close agreement between the values of the mean sizes directly assessed (TEM micrographs) and indirectly assessed (MTT data under illumination and dark conditions) is quite remarkable. Importantly, the morphological aspects of the nanomaterials have been screened directly (high resolution microscopies) and indirectly (selected standard experimental techniques, such as electrical, magnetic and optical) [44,45,46], with an agreement similar to our findings using TEM and MTT, the latter extracted from the Hill-inspired model herein proposed. It is expected that the values of $\langle s_{\mathrm{MTT}}\rangle$ and $\sigma_{\mathrm{MTT}}$, extracted from the fitting of the cell viability using the introduced Hill-inspired model will offer an alternative and indirect way to assess the morphological characteristics of NMs while incubated with cells, providing the key information about the herein introduced concept of “biological polydispersity”. It is worth mentioning that the mean biological sizes (illumination as well as dark) provided by the MTT assay are smaller than the value provided by the TEM micrographs. Although this finding requires more investigation, using different cell lines and the NM’s morphology and size range, it may point to the non-homogeneous distribution of binding sites between the flat circular surface of the F-GQD sample and the cell membrane, likely favoring the border region of the F-GQD instead of the center region, rendering a slightly reduced effective biological binding area and consequently reducing the typical biological size. Interestingly, the difference in electronic states and density of carriers between the inner region of GQDs and the edges under light excitation and dark conditions, plus different edge configurations (armchair and zigzag) [36], are certainly behind the differences observed while comparing the morphological parameters extracted from TEM and MTT and reported in the present study. Moreover, it is very likely that the analysis presented here can be extended to different in vitro assays employing NMs. Secondly, the K parameters listed in Table 1 show that the dark value is about 15 times larger than the illumination value. As K scales with the dissociation constant (see text explaining Equation (1)) it means that illumination very much enhances the cytotoxicity of the F:GQD sample against the HepG2 cells, herein translated as the enhancement of the F:GQD binding onto the cell membrane. In fact, this finding is very much consistent with the expected values of $\langle n\rangle =n_{0}+\alpha \left(\langle s\rangle -s_{0}\right)$, estimated from the parameters collected in Table 1, which were about 28% larger for the illumination condition (〈n〉≅2.3) compared to the dark condition (〈n〉≅1.8). This finding is a strong indication of the promising application of the F:GQD structure in PDT [47].

The colored solid lines in Figure 1a,b are simulations using Equation (4) inputted with the parameters listed in Table 1 (K, $n_{0}$, α, $s_{0}$) while increasing 〈s〉 from 2.0 nm up to 3.5 nm and fixing $\sigma_{\mathrm{MTT}}=0.21$ (illumination) and $\sigma_{\mathrm{MTT}}=0.22$ (dark), respectively. Analyses of the simulated data (colored solid lines) in Figure 1a,b suggest that the smaller F:GQD sample enhances cell viability, as the smaller (small 〈s〉 value) the mean size, the higher the cell viability in the whole range of doses evaluated. In terms of a model picture and considering the polydisperse F:GQD samples, the larger the mean size (〈s〉), the higher the mean number of binding sites (〈n〉), as these two variables are assumed to scale linearly ($\langle n\rangle =n_{0}+\alpha \left(\langle s\rangle -s_{0}\right)$). Note that this behavior is consistent with the outcomes of the original Hill equation (Equation (2)) from which the cell viability reduces more abruptly for larger values of *n* than for smaller values of *n* (see Equation (2)). Interestingly, as pointed out by Güçlü et al., regardless of the shape and edge termination, the energy gap of GQDs opens up monotonically as the size reduces, implying a higher energy absorption to promote carriers from the ground state up to an excited state, the latter much more reactive than the former [48]. This electronic characteristic of GQDs may shine some light on the results observed in the simulated curves collected in Figure 1a,b. Once excited, the carrier transfer between the GQDs attached onto the cells’ membranes becomes unfavorable as the NM’s size reduces, thus quenching possible redox processes. The observed trends in Figure 1a,b signal in favor of this argument, as the change in cell viability is much more pronounced under light illumination than in dark conditions. In regard to the monotonic cell viability reduction as the mean size increases, there are reports in the literature supporting the simulations herein included. Moreover, different in vitro assays using different cell lines also display a similar cell viability trend as far as the mean size of NMs are concerned, as listed in what follows. Wang et al. performed MTT assays with a human skin cell line (HaCaT keratinocytes) to assess the cell viability of polystyrenesulfonate-coated, rod-shaped Au-nanoparticles (5 nm, 12 nm and 30 nm in mean length) [49]. The authors observed a slight reduction in the cell viability as the rod length increased, while keeping roughly the same rod diameter. Using leukemia cancer cell lines (K562, K562/A02), Guo et al. conducted MTT assays to assess the cell viability of pristine, spherical ZnO-nanoparticles (20 nm, 60 nm and 100 nm in mean size) [50]. The authors found the IC50 value reducing as the mean particle size increased for both cell lines. Vedantam et al. performed MTS and cell uptake assays with prostate cancer cells (DU-145) to assess the cell viability and cell uptake of pristine and D-mannose-coated, spherical Au-nanoparticles (20 nm and 200 nm in mean size) [51]. The authors found the cell viability reduced as the mean size increased for pristine nanoparticles (NPs), in the cell log phase as well as in the cell lag phase. Moreover, cell (DU-145) uptake shows higher biocompatibility for the 20 nm NPs than for the 200 nm NPs, using pristine or protein-coated NPs. Best et al. conducted MTT and LDH assays, using oral epithelial keratinocytes (H376), to assess the cell viability and citotoxicity of pristine, rod-shaped ZnO-NPs (20 nm and 70 nm in mean length) [52]. The authors found that the cell viability systematically reduced as the NP rod length increased, while keeping roughly the same NP rod diameter. Likewise, LDH percent cytotoxicity systematically increased as the rod length increased. Using the human lung carcinoma cell line (A549) and red blood cells (RBC), Purohit et al. performed MTT and hemolysis assays to assess the cell viability and percentage of hemolysis of pristine (18 nm, 39 nm, 52 nm and 76 nm in mean size) and BSA-coated (31 nm, 48 nm, 70 nm and 136 nm in mean size) spherical Au-NPs [53]. The authors found hemolysis systematically increasing as the NP size increased; the increase was steeper for pristine than for BSA-coated. Moreover, in line with the hemolysis assay, cell viability decreased as the NP size increased, for both pristine and BSA-coated. Tippayawat et al. performed MIC assay, using gram-positive *S. epidermidis* (ATCC35984) and gram-negative *P. aeruginosa* (ATCC27803), to assess the antibacterial activity of (aloe vera)-coated spherical Au-NPs (95 nm, 150 nm and 192 nm in mean size) [54]. The authors found that the inhibition zone diameter (IZD) systematically increased as the NP size increased, for both pathogenic bacteria strains. Using two human hepatoma cell lines (SK-Hep-1 and Hep3B) Xie et al. performed mitochondrial activity, induction of ROS and induction of apoptosis and necrosis assays to assess the size-dependent cytotoxicity of pristine, spherical magnetite-NPs (6 nm, 9 nm and 14 nm in mean size) [55]. For both cell lines, at all NPs concentration used, the authors found the mitochondrial function of the 6 nm NP higher than the 9 nm or 14 nm NPs. Additionally, in line with the mitochondria function assay, for both cell lines, at all NP concentration used, induced ROS and induced apoptosis and necrosis assays revealed that the 6 nm NP was more biocompatible than the 9 nm or 14 nm NPs. Kang et al. reported MTT assay using Caco-2 cell line to assess the cell viability of chitosan-coated, spherical PLGA-NPs (165 nm, 261 nm, 337 nm and 481 nm in mean size), loaded with albendazole (ABZ) [56]. The authors reported that the cell viability of ABZ-loaded PLGA-NPs systematically decreased as the NP size increased. Using the HeLa cell line and Gram-positive *M. tuberculosis* and Gram-negative *Salmonella* strains, Pasha et al. performed MTT and antimicrobial assays to assess the cell viability and the antibacterial activity of pristine, spherical iron sulphide/bismuth oxide-NPs (59.6 nm, 61.4 nm and 63.6 nm in mean size) [57]. The authors found that the cell viability decreased as the NP size increased from 59.6 nm to 61.4 nm. In line with this finding, the IZD systematically increased as the NP size increased, for both pathogenic bacteria strains. Using Hepa 1-6 cancer cell line Madlum et al. conducted MTT assay to assess the cell viability of pristine, spherical Pt-NPs (10 nm and 20 nm in mean size) [58]. The authors found that the cell viability decreased as the NP size increased from 10 nm to 20 nm.

In a different way, the analysis of the simulated data (colored solid lines) in Figure 2a suggests that a higher polydispersity enhances cell viability under illumination conditions, as the more polydisperse (larger σ value) the sample, the higher the cell viability in the whole range of doses evaluated. In contrast, the analysis of the simulated data (colored solid lines) in Figure 2b suggests that a lower polydispersity enhances cell viability under dark conditions, as the more polydisperse (larger σ value) the sample, the lower the cell viability in the whole range of doses evaluated. Note, however, from Figure 2a,b, that the cell viability variation is much more sensitive to the sample’s polydispersity under illumination than under dark conditions. Explanation for the higher sensitivity of cell viability under illumination conditions, as observed in Figure 2a while compared with Figure 2b, may rely on the same model picture introduced above when analyzing comparatively the simulation curves presented in Figure 1a,b. It is worth stressing that the influence of the sample’s polydispersity in the extreme small range of mean size values (smaller than 4 nm) is not only reduced in the dark condition, as shown by the vertical scale in Figure 2b, but also in the opposite direction with respect to the trend observed in the illumination condition (see Figure 2a). In this regard, the proposed model picture for the cell viability (V) dependence on the size dispersity (σ) starts with the observed behavior of the cell viability (V) dependence on the mean size (〈s〉), as revealed in Figure 1a,b. Importantly, from the phenomenological point of view the simulated curves in Figure 1a,b show the cell viability scaling with the inverse of the mean size (V~1/〈s〉). A more general expression for such a behavior can be written as $V\sim p^{a}/{\left(\langle s\rangle \right)}^{p-a}$, with p and a representing parameters to be fitted (inputted) with experimental (simulation) data. The rate at which the cell viability changes with respect to the mean size (ΔV/Δ〈s〉) can be estimated by $\Delta V/\Delta \langle s\rangle \sim \left(a-p\right)p^{a}/{\left(\langle s\rangle \right)}^{p-a-1}$. In fact, Δ〈s〉 scales with σ (one takes Δ〈s〉~σ) and, therefore, the increment in cell viability (ΔV) can be written as $\Delta V\sim \sigma \left(a-p\right)p^{a}/{\left(\langle s\rangle \right)}^{p-a-1}$. It is worth mentioning that the simulations collected in Figure 1a,b impose that p−a−1>0, i.e., a−p<1. Two distinct solutions emerge from the a−p<1 condition, namely (i) 0<a−p<1 and (ii) a−p<0. The first case (i) leads to positive values for the increment in cell viability (ΔV) as the size dispersity (σ) increases, thus accounting for the simulations presented in Figure 2a. In contrast, the second case (ii) leads to negative values for the increment in cell viability (ΔV) as the size dispersity (σ) increases, thus accounting for the simulations presented in Figure 2b. The transition from one scenario to another, i.e., from the first case (i) to the second case (ii) depends on the experimental condition, meaning the parameters p and a, herein represented by illumination or dark experimental conditions. Although the presented model picture is phenomenological, it accounts for the trends observed in the simulations, which started with the fitting parameters of the experimental cell viability data extracted from the MTT assays. Last, but not least, reports on the cell viability of NMs at increasing size dispersity, while fixing the mean size, were not found in the literature. Our finding regarding the trends in cell viability versus size dispersity may represent an important stimulus for planning future experiments.

## 5. Conclusions

The present report provides a pioneering contribution to studies on the inclusion of the polydispersity of nanomaterials (mean size and size dispersity) while under in vitro evaluation for binding to cells and biomolecules using the original Hill model as the starting point. The Hill-inspired model herein proposed takes the Hill cooperativity index (n) scaling linearly with the typical size (s) of the nanomaterial (NM). Moreover, a lognormal distribution function was proposed for averaging out the cell viability (V), thus including the NM’s mean size (〈s〉) and size dispersity (σ) into the Hill’s equation. The proposed Hill-inspired model was successfully used to fit cell viability assessed from MTT assays and further used the extracted parameters to simulate cell viability under two different conditions: in the dark and under illumination. Simulations were based on MTT data, where HepG2 cells were challenged with fluorine-containing graphene quantum dots (F:GQDs). Additionally, in each of the two simulations the influence of the mean size (〈s〉) and size dispersity (σ) was evaluated in the range of 2.0–3.5 nm and 0.25–0.40, respectively. Under the two experimental conditions evaluated (dark and illumination), the observed simulations showed that at fixed size dispersity the cell viability monotonically decreases as the mean size increases. Differently, while fixing the mean size, the performed simulations resulted in two opposite trends phenomenologically accounted for by $V\sim p^{a}/{\left(\langle s\rangle \right)}^{p-a}$: (*i*) under illumination the cell viability increases as the size dispersity increases and (*ii*) in the dark the cell viability decreases as the size dispersity increases. The two opposite trends observed in the simulations of cell viability versus size dispersity were accounted for by a phenomenological model picture, in which the monotonic reduction of V as 〈s〉 increases leads to two opposite behaviors for V versus σ. Importantly, the reported electronic structure of GQDs, with wide open band gap energy in the extreme lower size range, points to the reduction in cytotoxicity of very tiny structures, as observed in the simulated curves. Indeed, the present report offers a pioneering and sound Hill-inspired model accounting for the size influence, as far as the biological response is concerned while testing nanomaterials. Importantly, the Hill-inspired model offers the opportunity for a comprehensive evaluation of the size effect of nanomaterials, such as the comparison between the “biological polydispersity” ($\langle s_{\mathrm{MTT}}\rangle$ and $\sigma_{\mathrm{MTT}}$) assessed from a standard cell viability assay (MTT) and the morphological polydispersity ($\langle s_{\mathrm{TEM}}\rangle$ and $\sigma_{\mathrm{TEM}}$) assessed from high resolution microscopy (TEM). Finally, it is herein anticipated that the present Hill-inspired model could be straightforwardly adapted to account for a variety of in vitro assays while testing nanomaterials.

## Figures and Tables

**Table 1:** List of parameters and statistical indexes extracted from the curve fit of the cell viability (MTT assays) using Equation (4), in the experiments under illumination and dark conditions.

**Parameters**	**Illumination**	**Dark**
K	(3.2 ± 0.5) × 10^4^	(4.7 ± 0.5) × 10^5^
$n_{0}$	1.97 ± 0.03	2.20 ± 0.02
α (nm^−1^)	1.4 ± 0.2	1.0 ± 0.2
$s_{0}$ (nm)	1.55 ± 0.02	2.27 ± 0.02
$\langle s_{\mathrm{MTT}}\rangle$ (nm)	1.77 ± 0.02	1.87 ± 0.02
$\sigma_{\mathrm{MTT}}$	0.21 ± 0.02	0.22 ± 0.01
**Statistics ^1^**	**Illumination**	**Dark**
Sum of squared deviations	0.0049	0.0005
R-squared	0.9984	0.9999
Correlation	0.9893	0.9805

^1^ Scientist™ MicroMath^®^.
